# Mechanical Behavior of Blood Vessels: Elastic and Viscoelastic Contributions

**DOI:** 10.3390/biology10090831

**Published:** 2021-08-26

**Authors:** David Sánchez-Molina, Silvia García-Vilana, Jordi Llumà, Ignasi Galtés, Juan Velázquez-Ameijide, Mari Carmen Rebollo-Soria, Carlos Arregui-Dalmases

**Affiliations:** 1 Escola d’Enginyeria de Barcelona Est, Universitat Politècnica de Catalunya, Av. Eduard Maristany, 16, 08019 Barcelona, Spain; silvia.garcia.vilana@upc.edu (S.G.-V.); jordi.lluma@upc.edu (J.L.); juan.velazquez@upc.edu (J.V.-A.); 2 Institut de Medicina Legal i Ciències Forenses de Catalunya, G.V. Corts Catalanes, 111, 08014 Barcelona, Spain; ignasigaltes@gmail.com; 3 Departament de Psiquiatria i de Medicina Legal, Universitat Autònoma de Barcelona, Cerdanyola del Vallès, 08193 Barcelona, Spain; 4 Instituto de Medicina Legal de Aragón, Irene Izárbez, 22005 Huesca, Spain; mcrebollos@aragon.es; 5 Centro Zaragoza, Crtra. 232, km. 273, 50690 Pedrola, Spain; carlos.arregui@centro-zaragoza.com

**Keywords:** biomechanics, collagenous tissue, tissue characterization, strain rate dependent materials, viscoelasticity

## Abstract

**Simple Summary:**

A frequent type of injuries in traffic collisions and falls from a moderate height is associated with subdural hematomas caused by the mechanical failure of cerebral bridging veins, which link the superior sagittal sinus to the brain. For this reason, both to design safe restraint systems for motor vehicles and to study how these injuries occur, it is important to study the mechanical properties of the bridging veins. Although the mechanical properties of bridging veins have been studied for the last half century, some viscoelastic effects in these vessels that alter their mechanical response have not been analyzed in detail until now. This is the first study that measures, quantifies, and models these viscoelastic effects, thus improving our knowledge of the mechanical response of cerebral bridging veins.

**Abstract:**

The mechanical properties of the cerebral bridging veins (CBVs) were studied using advanced microtensile equipment. Detailed high-quality curves were obtained at different strain rates, showing a clearly nonlinear stress–strain response. In addition, the tissue of the CBVs exhibits *stress relaxation* and a *preconditioning effect* under cyclic loading, unequivocal indications of viscoelastic behavior. Interestingly, most previous literature that conducts uniaxial tensile tests had not found significant viscoelastic effects in CBVs, but the use of more sensitive tests allowed to observe the viscoelastic effects. For that reason, a careful mathematical analysis is presented, clarifying why in uniaxial tests with moderate strain rates, it is difficult to observe any viscoelastic effect. The analysis provides a theoretical explanation as to why many recent studies that investigated mechanical properties did not find a significant viscoelastic effect, even though in other circumstances, the CBV tissue would clearly exhibit viscoelastic behavior. Finally, this study provides reference values for the usual mechanical properties, as well as calculations of constitutive parameters for nonlinear elastic and viscoelastic models that would allow more accurate numerical simulation of CBVs in Finite Element-based computational models in future works.

## 1. Introduction

Worldwide, TBI contributes to more fatalities and disabilities than any other traumatic event, with an average incidence of about 940 cases per 100,000 people; thus, between 64 and 74 million people suffer some form of TBI each year [1]. TBI is also the main traumatic cause of severe injuries and death in traffic collisions, and it can also occur in falls from a moderate height. For this reason, a good understanding of the injury mechanisms involved in TBI could be important to prevent them or to improve their medical treatment.

Among the injury mechanisms that can produce a serious TBI, damage to cerebral brain vessels is especially frequent. The incidence in all non-missile head injuries ranges from 26% to 63% [2,3], and the mortality rate ranges from 30% to 90% [3,4]. In addition, this damage is extremely life threatening since the mechanical failure of these vessels often produces subdural hemorrhages of some consideration and poses a serious risk to the neural tissue. The consequences of such injuries are dysfunctions of the vasculature, chronic neurodegenerative consequences [5], disabilities [6,7], and fatalities [8].

Regarding blood vessel damage, it is a well-known fact that an excessive tensile strain in certain vessels, such as CBVs departing from the sagittal sinus towards the encephalic mass, frequently results in a loss of structural integrity, leading to hemorrhage and SDH [9]. Such CBV damage can be produced by strong head decelerations or angular accelerations during a traumatic event. Another important issue is the age effect on the mechanical response in both the pediatric case [10,11] and in the elderly case [12], Due to this, obtaining further knowledge about the biomechanics of these injuries is important in the field of forensic pathology and neuropathology. More specifically, in cases of elderly or infant cranio-encephalic trauma, where it is important to differentiate between accidental and intentional trauma [13].

The mechanical properties of CBVs have been studied for more than half a century [14,15], mainly through uniaxial tensile tests due to the small dimensions and geometrical characteristics of CBVs. The results of most researchers are comparable and can be found in some excellent recent review articles on the mechanical properties of CBVs [16]. Interestingly, most recent works agree in suggesting that there is no significant viscoelastic effect in the CBVs [3,17,18,19,20,21] since YM do not increase significantly with the strain rate. Nevertheless, our study demonstrates that, although the viscoelastic effect is apparently negligible in uniaxial tests, in relaxation or repeated load-unload tests as performed, the viscoelastic characteristics are clearly measurable. In fact, the apparent absence of viscoelasticity in uniaxial tensile tests observed in previous studies is theoretically explained in the present research. Although it appears that viscoelastic behavior does not exert a major influence in material response [16] and the mechanical behavior is fairly well represented by an elastic model, an accurate characterization of the mechanical response of collagenous tissues is critical for investigating soft tissue injury mechanisms [22]. Therefore, after verifying the presence of viscoelastic effects, a viscoelastic model is used to quantify the weight of viscoelasticity in the mechanical behavior of CBVs. The viscoelastic model describes the mechanical behavior better than the known elastic models, and furthermore, it allows quantifying the viscoelastic contribution in CBVs, which will show that, indeed, it is really not negligible.

Thus, given the importance of the failure of CBV for SDH, the aim of this study is to improve the current state of knowledge on the mechanical behavior of CBVs. In addition, our work could help determine more accurate ranges of stress and force involved in the occurrence of an SDH, which is an important matter in forensic reconstruction.

## 2. Materials and Methods

### 2.1. Material and Specimen Preparation

The material used in this study consisted of a sample of human CBV specimens, harvested from forensic autopsies, conducted at the Forensic Pathology Service of the Legal Medicine and Forensic Science Institute of Catalonia (FPS/IMLCFC). The study was approved by the Research Committee of the IMLCFC.

For the tests, twelve sections of the meningeal-cortex space (including the meninges, the subarachnoid space, and the upper part of the cerebral cortex) were obtained from autopsies of N=12 PMHSs. Cases were limited to those where no cerebral vessel pathology was previously diagnosed. Once received, the sections were kept refrigerated for at most 96 h in airtight containers that maintained the natural degree of hydration.

**Figure 1:**
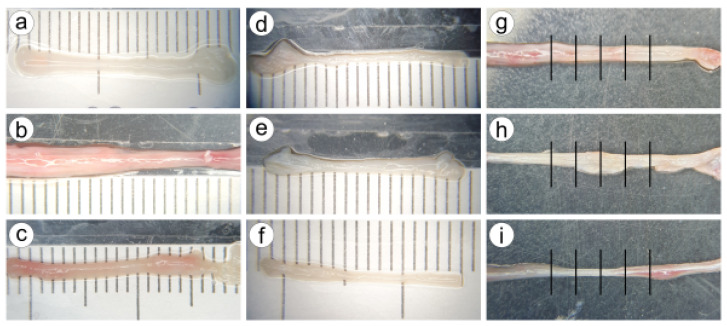
Nine images of different CBV specimens captured with the camera coupled to the microscope (**a**–**i**). The diameter and width vary slightly along the CBV. Subfigures (**g**–**i**) show the five measures of the apparent width in the central region. This was repeated for all CBVs, so the outer diameter OD was computed from measures of the width.

From these n=23 sections, CBVs were carefully dissected and tested in uniaxial tensile tests (more than one CBV was dissected from some sections). Each CBV was photographed using a camera coupled to a stereo microscope SMZ-168, both from Motic^®^ (see Figure 1). A scale was located under the CBV to measure the flattened CBV width. For each CBV, five width measures were taken in the central region (see Figure 1), and from the average of the five measures, the outer diameter (OD) was calculated. On the other hand, the thickness (*e*) was determined using the expression e=0.021+0.0061·OD as a function of the diameter, given in the literature [17]. Thus, the average area of the cross-section of each CBV could be determined from OD and thickness.

### 2.2. Mechanical Tests, Measures of Strain and Stress

The tensile tests, the cyclic load and relaxation tests were performed with a UTM Zwick-Roell^®^ (model: Allround-Table-Top^®^), and the applied load was measured with a 20 N load cell HBM^®^. Special fixtures were used, and the displacement was carefully measured with a digital control unit attached to the UTM. The fixtures had a knurled surface in order to prevent any slippage: accurate measures of the initial and final positions made it possible to verify whether there was no slippage inside the special fixtures.

*Uniaxial tensile tests*. The strain rate of uniaxial tensile tests ranged from $\dot{\varepsilon }$ = 0.001 to 1.20 s${}^{-1}$ (see Table 1). The final elongation was around 50% for many specimens and, because of this, the infinitesimal strain theory was not suitable, and finite strain measures were used instead.

As in all previous studies, each CBV is then considered a hollow cylinder of a constant cross-section and made of a homogeneous and transverse isotropic material [3,9,20], stretched along its longitudinal axis. Therefore, the longitudinal stretch for each instant $\lambda_{t}$ was computed as:

(1) $$\lambda_{t}=1+\frac{\delta_{t}}{L_{0}}$$

where $\delta_{t}$ is the digitally measured displacement and $L_{0}$ the undeformed length of the specimen. For the computation of strain and stress components, the origin of coordinates was located on the static fixture, and the X axis was chosen parallel to the stretching direction, while the Y and Z axes were parallel to the cross-section of the vein. Thus, the deformation of the cylinder can be described by relating explicitly the spatial coordinates $x_{t}=\left(x_{t},y_{t},z_{t}\right)$ of the current deformed or stretched configuration to the material coordinates $X_{t}=\left(X_{t},Y_{t},Z_{t}\right)$ of the initial undeformed configuration, by means of the following relations:

(2) $$x_{t}=X\lambda_{t},\;y_{t}=Y{\left[1-\bar{\nu }\left(\lambda_{t}^{2}-1\right)\right]}^{1/2},\;z_{t}=Z{\left[1-\bar{\nu }\left(\lambda_{t}^{2}-1\right)\right]}^{1/2}$$

where $\bar{\nu }$ considers the *Poisson effect*: for a nonlinear material, this coefficient is a function of the stretch, and when the material is linear elastic, the function $\bar{\nu }\left(\lambda_{t}\right)$ reduces to a constant. For the chosen constitutive model, the specific form of the function $\bar{\nu }$ is obtained in Section 2.3.1. On the one hand, the deformation gradient tensor $F=\partial x_{t}/\partial X$ can be determined from Equation (2):

(3) $$F=\left[\begin{array}{ccc} \lambda_{t} & 0 & 0\\ 0 & {\left[1-\bar{\nu }\left(\lambda_{t}^{2}-1\right)\right]}^{1/2} & 0\\ 0 & 0 & {\left[1-\bar{\nu }\left(\lambda_{t}^{2}-1\right)\right]}^{1/2} \end{array}\right]$$

Furthermore, the Green–Lagrange strain tensor is given by $E=(F^{T}F-1)/2$ as:

(4) $$E=\frac{1}{2}\left[\begin{array}{ccc} \lambda_{t}^{2}-1 & 0 & 0\\ 0 & -\bar{\nu }\left(\lambda_{t}\right)\left(\lambda_{t}^{2}-1\right) & 0\\ 0 & 0 & -\bar{\nu }\left(\lambda_{t}\right)\left(\lambda_{t}^{2}-1\right) \end{array}\right]$$

On the other hand, the second Piola–Kirchhoff stress tensor S is related to the Cauchy stress tensor σ (defined on the deformed configuration) by the relation $S=JF^{-1}\sigma F^{-T}$, where J=det(F) is the Jacobian determinant [23]. Explicitly, in components:

(5) $$S=\left[\begin{array}{ccc} \frac{F_{t}}{\lambda_{t}A_{0}} & 0 & 0\\ 0 & 0 & 0\\ 0 & 0 & 0 \end{array}\right]$$

where $F_{t}$ is the tensile force at time *t*, and $A_{0}$ is the initial cross-section of the specimen. This tensor takes into account the section variation due to stretching, which is given by:

(6) $$A_{t}=A_{0}\left[1-\bar{\nu }\left(\lambda_{t}^{2}-1\right)\right]$$

**Figure 2:**
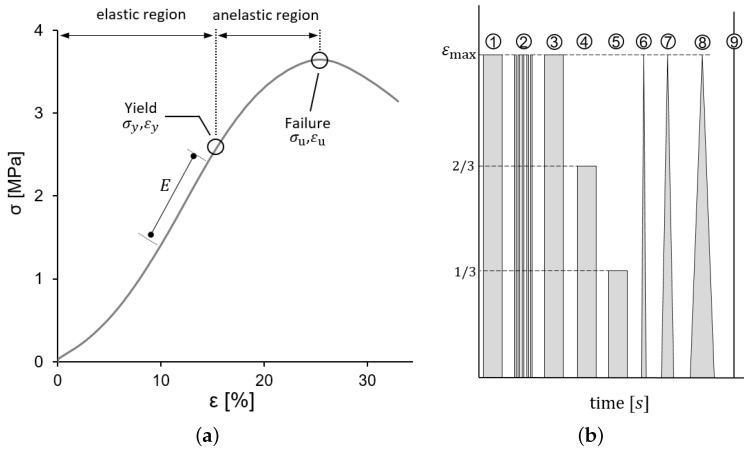
(**a**) Typical Stress–Strain curve obtained for CBVs showing the characteristics determined; these parameters were proposed in [20] and subsequently used also in [3,9]. (**b**) The scheme of the different stages of the testing procedure (relaxation, cyclic loading and loading-unloading tests).

Figure 2a shows a typical stress–strain curve of one of the CBVs tested and the main features measured from the data. The elastic region (with reversible deformation) and anelastic region (with non-reversible deformation) are separated by the yield point, defined by its stress $\sigma_{y}$ and strain $\varepsilon_{y}$, where the stress–strain curve changes its curvature from convex to concave. The maximum stress $\sigma_{u}$ defines the failure point, related to a failure strain $\varepsilon_{u}$. All those parameters were computed, together with YM, which was determined as the local slope of the curve at strain levels 10% and 15% to facilitate a comparison with the literature [16].

*Additional mechanical tests*. Furthermore, to verify the existence of measurable viscoelastic effects, relaxation and cycling loading tests were performed in three additional specimens. The testing process was divided into different stages, as illustrated in Figure 2b, following a process as in [24]. In these additional tests, the maximum strain applied was chosen as the 20% of the ultimate strain $\varepsilon_{u}$ of the CBVs tensile tests, corresponding to approximately a $\varepsilon_{u}=5\%$ of elongation. The different stages of Figure 1b consisted of: (1) loading-holding at $\varepsilon_{u}$, (2) a cyclic loading and unloading of 10 fast loading cycles, (3–5) three successive loading-holding tests at 3/3, 2/3 and 1/3 of $\varepsilon_{u}$, respectively, (6–8) three triangular loading-unloading stages at rates of 1, 0.1 and 0.01 s${}^{-1}$, respectively, and (9) load to failure. All strain jumps, in stages (1–5), were made at 3000 mm/min. In all relaxation tests and between loading stages, the CBV specimen was allowed to recover at no load for the full relaxation.

As will be presented in later sections, the results of these additional tests show a stress relaxation in the holding stages (1) and (3–5) and a progressive drop in the maximum CBV force between consecutive load-unload cycles of stage (2), being both phenomena characteristic of viscoelastic materials (the results are detailed and analyzed in Section 4). These effects could be observed by means of a very accurate digital force measurement, which allows observing the specimens that experienced a stress relaxation of the type expected for viscoelastic materials, as it is reported in the results section, showing that the viscoelastic effect, which was barely perceptible in the uniaxial tensile tests, was clearly observable in these complementary relaxation tests. This finding motivated the use of a viscoelastic model for the description of the mechanical behavior of CBVs in tensile tests, which have been previously considered in the literature as a material with elastic behavior. A viscoelastic model, such as those proposed in the next section, besides representing the behavior of CBVs better than an elastic model, allows to quantify the viscoelastic contribution in tensile tests, which is expected to be small, thus explaining why viscoelasticity is undetectable in this type of tests.

Furthermore, in the cyclic loading test stage, the final strain was always the same, and a decreasing trend in the force reached at successive peaks was observed. This experimental finding is attributed to the fact that the sample underwent preconditioning of its fibers in each subsequent cycle [24] so that in each cycle, the specimen exhibited a slightly higher stiffness than in the previous one; a result that fully coincides with what can be expected from viscoelastic behavior as modeled by a QLVE model.

The positive result for viscoelastic effects in these two additional tests led us to re-analyze the previous results of the uniaxial tensile tests by means of viscoelastic models. Therefore, this study compares the constitutive parameters obtained under the assumption of purely elastic behavior with that of viscoelastic behavior. As it is shown in Section 3, the viscoelastic contribution in the uniaxial tensile test is small but measurable.

### 2.3. Fung Models for Elastic and Viscoelastic Behavior

#### 2.3.1. Elastic Fung Model

**Figure 3:**
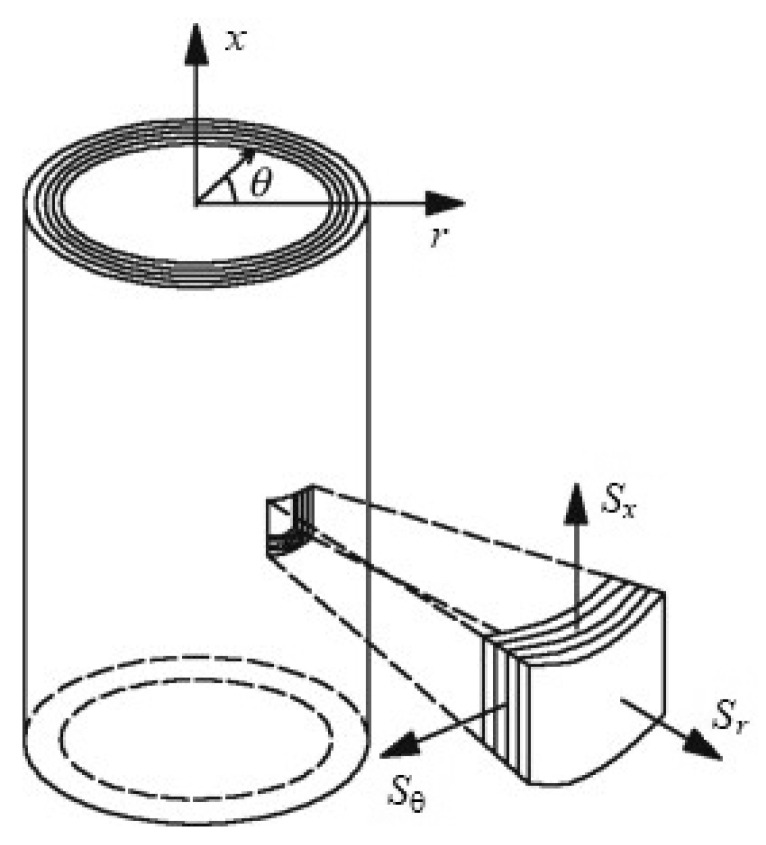
Adapted cylindrical coordinates used for the application of the Fung Model to a tubular section of the CBV.

Many collagenous soft tissues present a convex strain–strain curve, as shown in Figure 1a, in which the slope of the curve increases with the level of strain. In the literature, this feature is frequently modeled by means of “exponential-type” models. For the uniaxial case, this leads to an exponential relation of the form:

$$\sigma \left(\varepsilon \right)\propto e^{a\varepsilon^{2}}$$

where σ is the uniaxial stress measure, ε the strain measure, and a>0 a constant. Some models with these characteristics are the Fung–Deng model [25,26], the Holzapfel–Kroon model [27,28] or the Natali–Gregersen model [29], among others [30,31]. These models vary in complexity; all of them are a generalization of the classical hyper-elastic model of Fung [32], whose SEDF is a function of a function of the Green–Lagrange Strain Tensor E and is given by:

(7) $$\Psi \left(E\right)=\frac{c}{2}\left(e^{Q(E)}-1\right),\;Q\left(E\right)=b_{1}E_{x}^{2}+b_{2}E_{\theta }^{2}+b_{3}E_{x}E_{\theta }$$

where the auxiliary function Q(E) is a quadratic form of the components of the strain tensor. The strain components of the latter formula are expressed in the coordinate system of Figure 3. Equation (7) above shows that the model includes four constitutive parameters $c,b_{1},b_{2}$ y $b_{3}$, leading to a transverse isotropy of the vessel wall material. The transverse isotropy of blood vessels is discussed in detail in previous studies [27,28,33].

For the case of uniaxial stretching, it follows from Equation (4) that the nonzero components of the strain tensor are $E_{x}=\left(\lambda_{t}^{2}-1\right)/2$ and $E_{\theta }=E_{r}=-\bar{\nu }\left(\lambda_{t}^{2}-1\right)/2$. The elastic constitutive equations are obtained by calculating the following derivatives:

(8) $$\begin{array}{ccc} S_{x}^{\left(e\right)}=\frac{\partial \Psi }{\partial E_{x}} & =c\left(b_{1}E_{x}+\frac{b_{3}}{2}E_{\theta }\right)e^{Q} & =cE_{x}\left(b_{1}-\bar{\nu }\frac{b_{3}}{2}\right)e^{Q}\\ S_{\theta }^{\left(e\right)}=\frac{\partial \Psi }{\partial E_{\theta }} & =c\left(b_{2}E_{\theta }+\frac{b_{3}}{2}E_{x}\right)e^{Q} & =cE_{x}\left(-\bar{\nu }b_{2}+\frac{b_{3}}{2}\right)e^{Q}\\ S_{r}^{\left(e\right)}=\frac{\partial \Psi }{\partial E_{r}} & =0 \end{array}$$

Since in the case of uniaxial stress $S_{\theta }^{\left(e\right)}=0$, it follows from the second of these equations that $\bar{\nu }=b_{3}/2b_{2}$, so the Poisson effect, referred to in Equation (4), is given by a constant, expressible in terms of the original model constitutive parameters. Defining $b_{0}:=b_{1}-\bar{\nu }b_{3}/2=b_{1}-b_{3}^{2}/\left(4b_{2}\right)$, the first equation can be simplified to:

(9) $$S_{x}^{\left(e\right)}=cb_{0}E_{x}e^{b_{0}E_{x}^{2}}$$

This last equation is known as the *simplified exponential model*, which is equally hyper-elastic and can be derived from the following SEDF:

(10) $$\Psi \left(E,a\right)=\frac{c}{2}\left(e^{b_{0}a\cdot E^{2}\cdot a}-1\right)=\frac{c}{2}\left(e^{b_{0}E_{x}^{2}}-1\right)$$

The above equation constitutes a transverse isotropic hyper-elastic model in which a indicates the direction tangential to longitudinal direction of the CBV (which in the test coincides with the *X* direction). Since only uniaxial loading situations have been considered, in the following, we use the simplified exponential model (Equation (9)) to derive the rest of the relevant mathematical relations.

#### 2.3.2. Viscoelastic Fung Model

The most widely used viscoelastic model for soft collagenous tissues is the QLVE model, which was also proposed by Y.-C. Fung [32]. In this work, it is assumed as a first approximation that the viscoelastic effect can be adequately represented by a QLVE model, whose *relaxation function* $R(E_{x},t)$ is separable and factorizes as $R\left(E_{x},t\right)=G\left(t\right)S_{x}^{e}\left(E_{x}\right)$ [34]. In addition, given the short duration of the test, it is considered that G(t) can be approximated by a two-term Prony series $G\left(t\right)=\left(1+g_{1}e^{-t/\tau_{0}}\right)$, being $\left(g_{1},\tau_{0}\right)$ two additional constants of the QLVE model, called the *first viscoelastic coefficient* ($g_{1}$) and the *relaxation time* ($\tau_{0}$). The inclusion of additional terms in the Prony series does not fundamentally change the numerical results.

With these considerations, the second Piola–Kirchhoff stress is given by:

(11) $$\begin{array}{cc} S_{x}\left(t\right) & =\int_{0}^{t}G\left(t-\tau \right)\frac{\partial S_{x}^{\left(e\right)}}{\partial E_{x}}{\dot{E}}_{x}\left(\tau \right)\;d\tau \\ \; & =S_{x}^{\left(e\right)}\left(E_{x}\left(t\right)\right)+g_{1}b_{0}c\int_{0}^{t}e^{-\left(t-\tau \right)/\tau_{0}}\left[1+2b_{0}E_{x}^{2}\left(\tau \right)\right]e^{b_{0}E_{x}^{2}\left(\tau \right)}{\dot{E}}_{x}\left(\tau \right)\;d\tau \end{array}$$

Thus, the stress response $S_{x}$ of the material is the sum of the elastic $S_{x}^{\left(e\right)}$ and the viscoelastic $S_{x}^{\left(v\right)}$ responses, where the term containing the integral is the viscoelastic contribution to stress and is designated as $S_{x}^{\left(v\right)}\left(t\right)={\tilde{S}}_{x}^{\left(v\right)}\left(E_{x}\left(t\right),g_{1},\tau_{0},b_{0},c\right)$. In Appendix A, the explicit integration of Equation (11) for the constant strain rate is presented, and a detailed analysis of how the stress–strain curve varies in terms of the strain rate is also given.

Both the elastic model of Equation (9) and the viscoelastic model of Equation (11) were fitted to the tensile test results for CBVs to show the improvement of the viscoelastic model and compute the viscoelastic contribution and is exposed in the following sections.

## 3. Results

**Figure 4:**
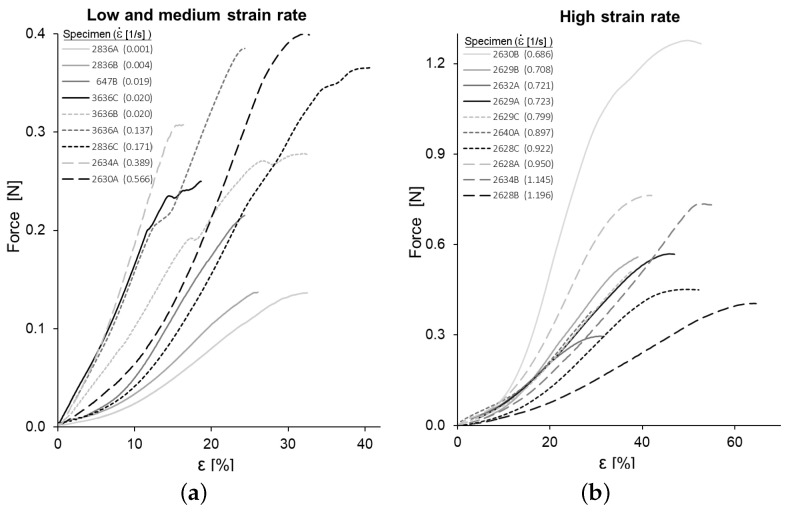
Force-strain curves of all specimens: (**a**) low and medium strain rate ($\dot{\varepsilon }<0.60$ s${}^{-1}$) and (**b**) high strain rate ($\dot{\varepsilon }>0.60$ s${}^{-1}$).

The force-strain curves obtained for the tensile tests are shown in Figure 4. The curves are clearly convex so that the effective YM at each strain level, measured as the local slope of the stress–strain curve, increases progressively. In this section, the strain ε is the longitudinal component of the *material Green–Lagrange Strain Tensor* and σ refers to the main stress of the *second Piola–Kirchhoff Stress Tensor*.

Table 1 contains all the values obtained for the *yield stress* ($\sigma_{y}$), the *ultimate stress* ($\sigma_{u}$), the *yield strain* ($\varepsilon_{y}$), the *maximum strain* ($\varepsilon_{u}$), the *effective* YM at strain levels ε = 10% ($E_{0.10}$) and ε = 15% ($E_{0.15}$), and the strain rate $\dot{\varepsilon }$ of each tensile test. Additional clarification of these parameters can be found in Figure 1. The specimen names in Table 1 and Table 2 consist of a number identifying the PMHS and the letter A, B, and so on, which is used when different specimens were obtained from the same PMHS. All the computed values for mechanical properties of CBV are close to the values found in similar studies [3,9,16,17,18,19,20], as discussed in Section 4.

The analysis of these data by means of MANOVA showed no significant correlation of the effective YM with the strain rate $\dot{\varepsilon }$, $E_{0.10}$ (*p*-value >0.90) y $E_{0.15}$ (*p*-value >0.60), as expected from the computations of Appendix A.

On the other hand, both the ultimate strain $\sigma_{u}$ (*p*-value <0.003) and the associate strain $\varepsilon_{u}$ (*p*-value <0.001) show a significant increase with the strain rate. Similarly, $\sigma_{y}$ (*p*-value <0.0001) and $\varepsilon_{y}$ (*p*-value <0.0001) are affected by the strain rate. Consequently, the maximum failure and elastic failure initiation forces are lower for specimens tested at a lower strain rate.

**Figure 5:**
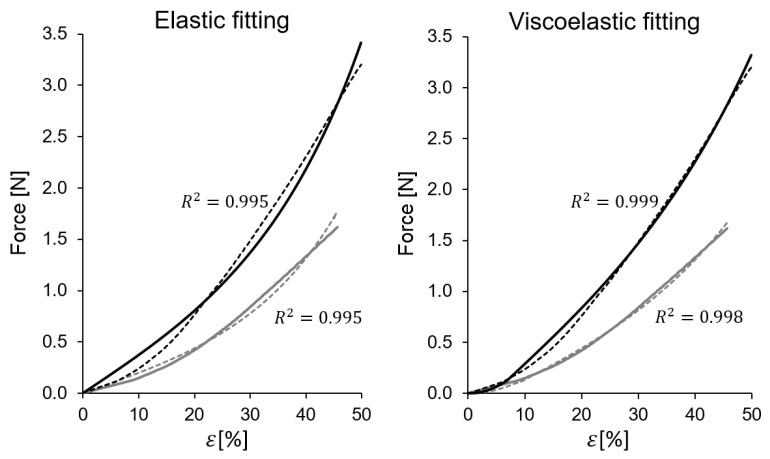
Two typical examples of force-strain curves, compared with the fitting of the elastic model and the fitting to the viscoelastic model (specimens 2628B (gray) and 2634C (black)).

For the stress–strain curves, both the elastic model of Equation (9) and the viscoelastic model of Equation (11) were fitted. Table 2 provides the parameter values of the fitted parameters for both models and additional information about the quality of the fitting ($r^{2}>0.97$ in all cases). The specimens in this table are arranged in order of increasing strain rate (SR) and classified in low SR, medium SR and high SR. A comparison of typical experimental stress–strain curves and fitted curves for both models is provided in Figure 5. From the elastic fitting the parameters, $b_{e}=b_{0}$ and $C_{e}=c\cdot A_{0}$ were computed, where $A_{0}$ is the gross area, and $c_{e}=C_{e}/A_{0}$ is the corresponding parameter for the stress–strain curve.

As it can be seen in Table 2 and in Figure 5, a purely elastic fitting based on Equation (9) is in all cases very good (${\bar{r}}^{2}=0.99$, on average). The fact that the elastic model provides such a good fitting could be the reason why many authors do not provide additional viscoelastic refinements since descriptively the elastic model already provides a reasonably good approximation of the stress–strain curves. However, as it is shown below, the graphical fit could be further improved with a more complex viscoelastic model.

In addition, due to the interest placed in this work in quantifying the viscoelastic contribution, a more complex model given by Equation (11) was considered. This allowed to quantify the weight of the viscoelastic effects. The viscoelastic model uses four parameters: $b_{0},c,\tau_{0}$, and $g_{1}$. Nevertheless, it was found that due to the mentioned short duration of most tests, the fitted value of the *characteristic time* $\tau_{0}$ exhibits high uncertainty, so the fitting procedure was repeated, keeping the parameter $\tau_{0}$ as 1,5,10 and 50 s. Within this value range of $\tau_{0}$, the value of the coefficient $g_{1}$ showed very little variation, so its value was robust despite the imprecision found in $\tau_{0}$. For that reason, the table shows only the fitted viscoelastic parameters $b_{v}=b_{0},c_{v}=c,g_{v}=g_{1}$ for each stress–strain curve. With the viscoelastic model, a graphical fitting improvement is seen with respect to the elastic model (Figure 5).

**Figure 6:**
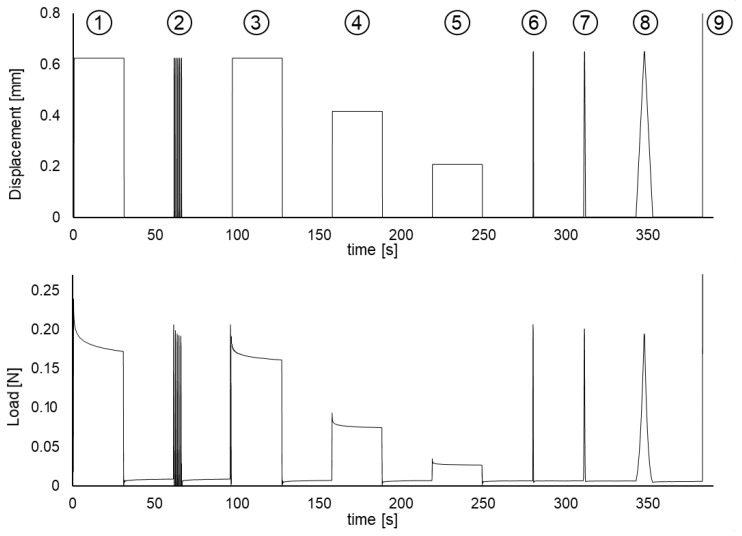
Mechanical tests showing viscoelastic effects: (1) initial relaxation test, (2) cyclic loading showing preconditioning, (3–5) repeated relaxation tests, (6–8) triangular wave loading, and (9) stretch to failure.

The additional cyclic loading and relaxation tests are summarized in Section 2.2 and Figure 1. Figure 6 shows the strain applied and the corresponding reaction force measured by the UTM. In the constant strain stages (1) and (3–5), it can be seen a stress relaxation of the force required to keep the strain constant. The stages (3–5) repeat the relaxation with different strain levels: 3/3, 2/3, and 1/3 of the strain used in stage (1). Moreover, in stage (2), fast cyclic loading is imposed to obtain a cyclic strain (period *T* = 0.50 s), and the result is that the successive force peaks are decreasing due to a viscoelastic preconditioning effect [24]. The observation of preconditioning and stress relaxation is incontrovertible evidence of viscoelastic behavior.

Therefore, the above results of the cyclic load and relaxation tests point to the existence of viscoelastic effects, supporting the use of a viscoelastic model, such as those proposed in (11), which makes it possible to explicitly quantify the viscoelastic contribution computing the weight of the viscoelastic effect. Moreover, this model could further improve the graphical fit given by the elastic model.

The viscoelastic model uses four parameters; $b_{0},c$, the *characteristic time*$\tau_{0}$, and $g_{1}$, which were obtained for all the tensile tests. Nevertheless, it was found that, due to the short duration of most tests, the obtained value of $\tau_{0}$ shows high uncertainty, being random and large; however, repeating different fittings for each test with different fixed values for $\tau_{0}=1,5,10$ and 50 s, the rest of parameters showed very little variation, being thus quasi-independent of $\tau_{0}$. Therefore, Table 2 shows the viscoelastic parameters $b_{v}=b_{0},c_{v}=c,g_{v}=g_{1}$ for each stress–strain curve, obtained for a range of $\tau_{0}$ between 1 and 50 s.

As it can be seen in Figure 5, the viscoelastic model improves the graphical fitting with respect to the elastic fitting, even the $r^{2}$ value increases slightly due to the good correlation already obtained with the elastic model.

The constitutive parameters of the elastic model do not show significant variations with the strain rate, as expected. On the other hand, in the viscoelastic model, the parameters $b_{v}$ and $g_{v}$ are not independent; in fact, they are negatively correlated, and $b_{v}$ decreases significantly with $\dot{\varepsilon }$ (*p*-value <0.011). The constitutive parameters in Table 2 seem to be distributed according to log-normal probability distributions; for this reason, the provided reference values are given in exponential form: $b_{e}=e^{2.19\pm 0.79}$ and $b_{v}=e^{1.87\pm 0.91}$ (or $b_{v}=e^{2.29-1.02\dot{\varepsilon }\pm 0.85}$). For modeling purposes, it is possible to use the formulation in terms of force F(ε) or stress σ(ε) since the cross-section of the FEHM is always known. In fact, many computational FEHM for CBV use 1D calculation schemes in such a way that the force parameters $C_{i}$ are directly used instead of the stress parameters $c_{i}=C_{i}/A_{m}$, where $A_{m}$ is the area of the cross-section of a specific vein of a FEHM. The reference values are $C_{e}=e^{-2.61\pm 1.09}$ and $C_{v}=e^{-2.69\pm 1.19}$. When the viscoelastic extension is used, the other reference value is $g_{v}=e^{-2.51\pm 1.26}$.

To determine how important the viscoelastic effect is in the mechanical behavior of CBVs subjected to a tensile test, the weight of the viscoelastic contribution (VC) was computed for each specimen, as:

(12) $$\mathrm{VC}=\frac{S_{x}^{\left(v\right)}\left(\varepsilon_{y}\right)}{S_{x}^{\left(e\right)}\left(\varepsilon_{y}\right)+S_{x}^{\left(v\right)}\left(\varepsilon_{y}\right)}=\frac{S_{x}\left(\varepsilon_{y}\right)-S_{x}^{\left(e\right)}\left(\varepsilon_{y}\right)}{S_{x}\left(\varepsilon_{y}\right)}$$

where the elastic $S_{x}^{\left(e\right)}$ and viscoelastic $S_{x}^{\left(v\right)}=S_{x}-S_{x}^{\left(e\right)}$ stresses are computed by means of Equation (11) and the values $b_{v},C_{v}$ and $g_{v}$ in the table. The VC exhibits a very significant increase with strain rate (p<0.003), which confirms the presence of viscoelastic effects, even in short-duration tensile tests.

## 4. Discussion

In this study, tensile tests of CBVs performed at different strain rates have been carried out to analyze the occurrence of viscoelastic effects in the mechanical response of CBVs. Additional cyclic load and relaxation tests were also carried on some CBVs to show how the viscoelastic contribution can be clearly seen in other tests different to tensile tests.

From the tensile tests, the mechanical properties of CBVs were obtained; the *yield stress* $\sigma_{y}=1.75\pm 0.98$ MPa, the *ultimate stress* $\sigma_{u}=2.62\pm 1.54$ MPa, the *yield strain* $\varepsilon_{y}=24.5\pm 10.9$%, the *maximum strain* $\varepsilon_{u}=35.3\pm 15.2$%, and the *effective* YM at strain levels ε = 10% and 15% $E_{0.10}=4.34\pm 1.32$ MPa and $E_{0.15}=5.67\pm 1.67$ MPa (see Table 1 for each specimen and Figure 1 for additional clarification of these parameters). All the above computed values are close to the values found in the literature on the mechanical properties of CBV [3,9,16,17,18,19,20]. In addition, the average values of geometrical dimensions of the sample in our study are OD =2.14±0.56 mm, e=0.034±0.003 mm, and $A_{0}=0.231\pm 0.086$ mm${}^{2}$. A comparison of the values of our study with the values are of other authors: Monson (2005) 1.84±0.35 mm [19], Delye (2006) 2.7±0.85 mm [20], Han et al. (2007) 2.5±1.1 mm [35], and Monea (2014) 3.42±1.18 mm [3], showing that all these data are comparable.

In our study, the strain rate ranged from 0.001 to 1.196 s${}^{-1}$. Additionally, a MANOVA analysis showed that the effective YM did not vary significantly with strain rate, in line with the findings of other research studies [3]. On the other hand, both the ultimate strain $\sigma_{u}$ and the associate strain $\varepsilon_{u}$ increase significantly with the strain rate, as other authors found [3,9].

The mechanical behavior of CBVs has been extensively studied in the literature [3,9,16,17,18,19,20,21], although many studies neglect to explicitly quantify the weight of viscoelastic effects in the stress/force-strain curves. However, the relaxation and cycling tests performed in this study clearly show a viscoelastic effect on the CBV response, especially in stress relaxation stages under a constant strain where the force on the CBVs is progressively reduced.

The fact that relaxation and cyclic loading tests showed clear viscoelastic effects, motivated that, in addition to the nonlinear elastic model (Section 2.3.1), an additional nonlinear viscoelastic model was considered (Section 2.3.2). The use of the two models allowed quantifying the weight of the viscoelastic effects reflected in the measure VC (see Table 2). In fact, the elastic model already produced good fits ($r^{2}>0.97$ for elastic model and $r^{2}>0.99$ for viscoelastic model), but graphically, the viscoelastic model improved the fittings graphically, as shown in Figure 5. The use of a viscoelastic model is not only confirmed by the results obtained from cyclic and relaxing tests, which clearly showed the presence of viscoelasticity, but also corroborated by the VC, which increased with the strain rate. In fact, VC was 4.20 ±3.83% for low SR ($\dot{\varepsilon }\leq 0.02$), 17.9 ± 14.6% for medium SR, and 35.3 ±10.8% for high SR ($\dot{\varepsilon }>0.6$), being not negligible values and thus confirming the existence of a viscoelastic contribution to the mechanical behavior of CBVs, even in short tensile tests. This finding is important because most authors have so far described the mechanical behavior of CBVs as purely elastic given the good fit of this type of model in tensile tests, ignoring the fact that CBVs are viscoelastic, as this study seems to demonstrate.

**Figure 7:**
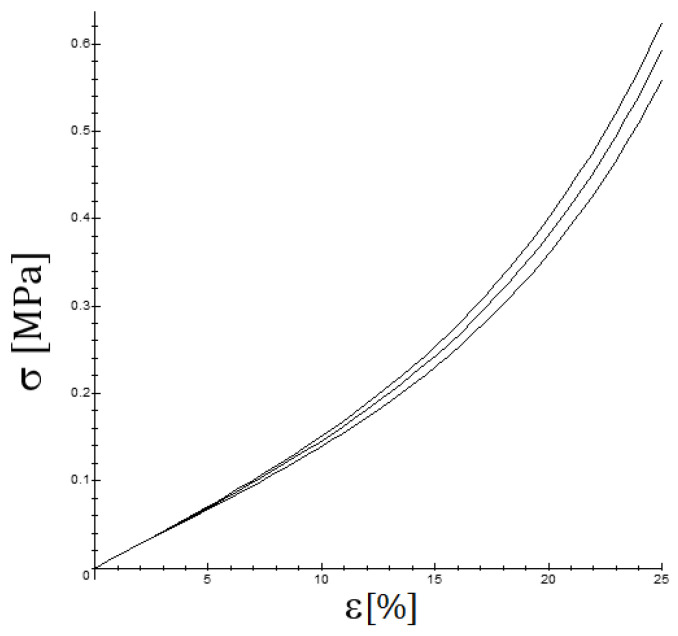
The influence of the strain rate on the stress–strain curve for $\dot{\varepsilon }=0.1/\tau_{0},0.2/\tau_{0}$ y $0.5/\tau_{0}$ (the upper curve occurs for the highest strain rate and the lower one for the lowest strain rate); the following values have been considered in the graph: $b_{0}=8$, c=0.10, $g_{1}=0.40$.

**Figure 8:**
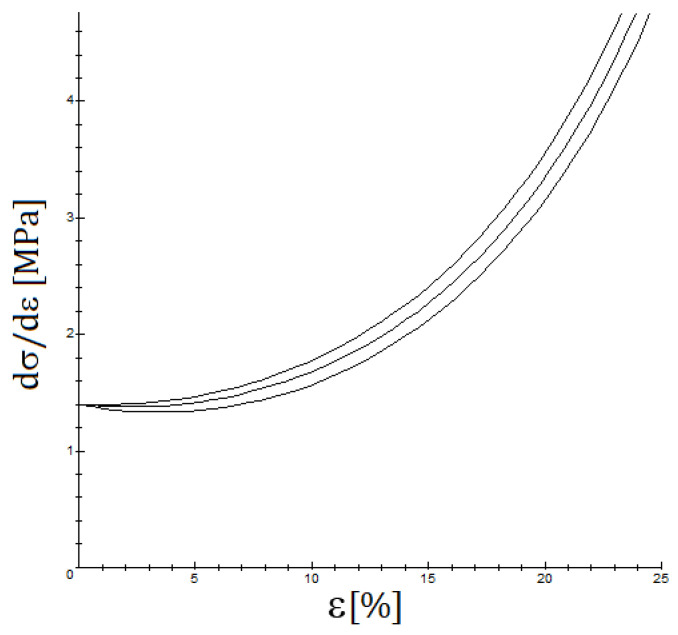
The influence of the strain rate on stiffness for $\dot{\varepsilon }=0.1/\tau_{0},0,2/\tau_{0}$ y $0,5/\tau_{0}$ (the upper curve is given for the highest and the lower curve for the lowest strain rate); the values used are $b_{0}=8$, c=0.10, $g_{1}=0.40$.

It is important to note that the most frequently used quasi-linear viscoelastic models [36,37], such as the standard Maxwell–Wiechert viscoelastic solid model (which is a generalization of the Maxwell model) and the Kelvin–Voigt model or the Burgers model (which generalizes it) predict that for a small strain, the YM should not vary significantly. For QLVE models containing a nonlinear part, such as the viscoelastic Fung model presented in Section 2.3, the computations are more complicated and are presented in Appendix A, but essentially the same conclusion is achieved, as is shown in Figure 7. The key observation is that the initial tangent Young’s modulus should not show a significant incidence of the strain rate, see Figure 8, when only viscoelastic effects are involved, just as several studies find [22,38]. However, at moderately high strain rates, the viscoelastic effect is measurable but small, and when the strain rate is low, the time for failure is longer, but the strain rate is low, and it is difficult to observe the viscoelastic effect. On the other hand, if a situation such as a relaxation test is presented or cyclic loading occurs, then the viscoelastic effect is more easily noticeable, see Figure 6.

With respect to nonlinear stress–strain response, it is interesting to note that some widely used computational models, such as early versions of SIMon, created by NHTSA, modeled CBVs as linear elastic cables rather than as cables with a nonlinear elastic response [39,40]. This would have a direct bearing on the estimation made in the injury metric called relative motion damage measure (RMDM) [41] used to predict the probability of SDH due to mechanical failure of some CBV [42,43]. Similarly, other human head computational models, among which are the UDS FEHM (Université de Strasbourg) [44], the KTH FEHM (S. Kleiven) [45], the UCDBTM (University College Dublin) [46,47], the WSUBIM (Wayne State University) [48], or the G/LHM [49], also model CBVs as elastic beams with a linear stress–strain response [16].

However, a new generation of FEHM consider the nonlinear behavior and viscoelasticity of brain structures, as it has been explicitly pointed out by the developers of YEAHM (University of Aveiro) [50]. Interestingly, some FEHM model CBVs as nonlinear elastic materials [51,52,53], and some of these models use curves and fittings based on [19,31]. These new models include geometric details of the vasculature and even consider the internal pressure inside the CBVs. With this level of detail, the viscoelastic contribution could play some role in the accuracy of the results because the reference values of our study incorporate, in addition to nonlinearity, the viscoelastic effects. Therefore, the work presented here has a potential application to some of the most recent FEHM, including a detailed geometry of the CBVs.

Another remarkable fact is that the peak force $F_{u}$ and failure onset force $F_{y}$, as well as the respective stresses $\sigma_{u}$ and $\sigma_{y}$, are lower for specimens with a lower strain rate or test velocities. This has also been found in other biological tissues [3,9,20,54] and could be related to an uneven distribution of the strain between fibers in collagenous tissue, although more work is needed to clarify the origin of this significant effect and found by several other authors.

Further work is needed in order to characterize full viscoelastic behavior in general situations. In such cases, a Prony series with more terms should be used for processes that extend over a longer period of time. On the other hand, due to the short duration of the tests and the fact that the desired strain level cannot be controlled, a simple QLVE model has been considered in which the relaxation function has been assumed separable, as explained in Section 2.3.2. However, a separable model of this type is not the most general model possible, although suitable for the type of restricted data analyzed here. The possible need to use more general non-separable viscoelastic models should be considered in future work.

## 5. Conclusions

The current study is the first published systematically comparing a nonlinear elastic model and a viscoelastic model of CBVs. This comparison allowed a quantification of the contribution of viscoelastic behavior, which critically depends on the strain rate. Moreover, the difficulty in observing viscoelastic effects at low strain levels has been explained theoretically. Accurate stress–strain curves have been obtained for this collagenous tissue, which can be used to assess TBI and SDH, improving the predicted material’s response. In addition, the reference values for the constitutive parameters of nonlinear constitutive models have been obtained, which could be used directly to make accurate computational FEHMs to assess TBI. In particular, the results of this study could be relevant to assess acute SDH due to the mechanical failure of CBV.

In addition, a significant positive correlation of the strain rate effect on the main ultimate stress (*p*-value <0.003) has been identified, while previous studies found inconsistent non-significant correlations. Other mechanical properties, such as the maximum strain, the yield stress, and the yield strain, also present a significant correlation with the strain rate (*p*-value <0.001, in all cases). Moreover, the effect of viscoelastic behavior has been quantified, being small in most situations, which explains why many applications could be modeled with a purely elastic model.

Finally, the results could be relevant for both of these approaches: the FEHM accuracy and the forensic reconstruction of injury mechanisms. In particular, the nonlinear stress–strain response of the CBVs has been described accurately using nonlinear elastic and viscoelastic models.

## Figures and Tables

**Table 1:** Basic mechanical properties of the CBV specimens.

Specimen	$E_{0.10}$	$E_{0.15}$	$F_{u}$	$\sigma_{u}$	$\varepsilon_{u}$	$F_{y}$	$\sigma_{y}$	$\varepsilon_{y}$	$\dot{\varepsilon }$
Identifier	(MPa)	(MPa)	(N)	(MPa)	(%)	(N)	(MPa)	(%)	($s^{-1}$)
2628A	4.56	6.60	1.036	3.73	42.0	0.650	2.34	26.7	0.950
2628B	2.10	2.37	0.612	3.38	64.6	0.405	2.24	45.6	1.196
2628C	3.18	4.29	0.643	3.95	52.2	0.383	2.35	31.9	0.922
2629A	5.03	6.51	0.791	4.44	46.9	0.426	2.39	27.7	0.723
2629B	4.88	7.02	0.746	4.06	39.0	0.479	2.60	27.4	0.708
2629C	5.11	6.36	0.680	3.89	37.9	0.533	3.05	31.7	0.799
2630A	3.66	5.60	0.513	2.17	32.8	0.349	1.47	23.6	0.566
2630B	4.74	8.57	1.814	5.00	52.7	0.902	2.49	24.3	0.686
2632A	5.63	6.80	0.378	2.32	31.8	0.378	2.32	31.8	0.721
2634A	6.10	—	0.278	0.65	16.3	0.204	0.47	13.3	0.389
2634B	2.59	2.83	1.060	3.17	55.0	1.036	3.10	51.2	1.145
2640A	5.94	6.95	0.876	5.17	52.8	0.581	3.43	32.9	0.897
2836B	2.05	3.18	0.175	1.08	32.5	0.108	0.66	21.7	0.001
2836C	4.02	6.65	0.091	0.79	18.3	0.091	0.79	18.3	0.004
2836D	3.80	6.09	0.493	3.03	41.0	0.241	1.48	22.9	0.171
3636A	4.48	4.86	0.470	1.34	24.3	0.313	0.89	17.4	0.137
3636B	4.92	5.43	0.357	1.60	32.6	0.238	1.07	19.1	0.020
3636C	6.61	—	0.293	0.93	18.7	0.213	0.68	11.3	0.020
647A	—	—	0.125	0.70	11.7	0.081	0.45	8.10	0.002
647B	3.10	6.36	0.262	1.01	24.3	0.164	0.63	17.2	0.019

**Table 2:** Fitted constitutive parameters for elastic and viscoelastic models.

		Elastic Model					Viscoelastic Model				
Specimen	$\dot{\varepsilon }$	$b_{e}$	$C_{e}$	$c_{e}$	$r^{2}$	$b_{v}$	$C_{v}$	$c_{v}$	*g*	$r^{2}$	VC
Identifier	(s${}^{-1}$)	(–)	(N)	(MPa)		(–)	(N)	(NPa)	(–)		(%)
2836B	0.001	14.92	0.015	0.091	0.991	14.92	0.015	0.091	0.000	0.991	0.04
2836C	0.004	17.70	0.016	0.140	0.997	17.70	0.016	0.141	0.000	0.997	0.00
647B	0.019	28.66	0.013	0.051	0.991	23.61	0.012	0.045	0.144	0.995	7.36
3636C	0.020	13.11	0.110	0.350	0.999	13.11	0.110	0.350	0.005	0.999	1.29
3636B	0.020	2.770	0.365	1.637	0.992	1.847	0.466	2.091	0.047	0.994	7.40
3636A	0.137	2.301	0.636	1.817	0.992	1.727	0.827	2.363	0.018	0.995	6.66
2836D	0.171	16.33	0.024	0.149	0.993	14.69	0.023	0.143	0.044	0.994	8.75
2634A	0.389	23.33	0.061	0.141	0.995	13.72	0.078	0.180	0.194	0.999	38.6
2630A	0.566	14.32	0.041	0.172	0.997	11.63	0.040	0.169	0.090	0.999	17.7
2630B	0.686	21.76	0.044	0.122	0.981	14.81	0.012	0.032	0.514	0.993	54.6
2629B	0.708	11.93	0.054	0.293	0.977	5.896	0.049	0.265	0.217	0.995	42.3
2632A	0.721	5.616	0.139	0.852	0.992	3.728	0.150	0.923	0.065	0.995	23.5
2629A	0.723	7.926	0.090	0.506	0.992	4.706	0.098	0.551	0.111	0.999	30.5
2629C	0.799	6.579	0.112	0.643	0.990	4.614	0.095	0.544	0.120	0.997	33.2
2640A	0.897	4.567	0.193	1.139	0.996	4.995	0.215	1.270	0.008	0.996	33.0
2628C	0.922	9.487	0.042	0.256	0.985	5.531	0.043	0.261	0.148	0.997	25.8
2628A	0.950	12.12	0.074	0.268	0.993	10.61	0.053	0.189	0.121	0.995	27.8
2634B	1.145	2.554	0.314	0.940	0.984	1.225	0.146	0.435	0.366	0.998	44.3
2628B	1.196	3.425	0.100	0.554	0.987	1.742	0.121	0.670	0.143	0.998	20.7

## Data Availability

Due to the restrictions imposed by the legal collaboration agreement between IMLFC and UPC, the data are not publicly available, although they can be accessed upon non-anonymous request to the authors of the study and under the conditions set by the aforementioned legal collaboration agreement.

## Abbreviations

The following abbreviations have been used in the text:

CBV(s)	Cerebral Bridging Vein(s)
FEHM	Finite Element Head Model
PMHS(s)	*Post Mortem* Human Subject(s)
QLVE	Quasi-Linear Viscoelastic
SDH	Subdural Hematoma
SEDF	Strain Energy Density Function
TBI	Traumatic Brain Injury
UTM	Universal Test Machine
VC	Viscoelastic Contribution
YM	Young’s modulus

## Appendix A. Viscoelastic Computations

Some analytical properties of Fung’s QLVE model are examined in this section. Explicit formulas are presented for different load cases, including the important case of constant strain-rate.

*Constant strain-rate case*. When the strain rate is constant, i.e., $E_{x}\left(t\right)=\beta_{0}t$ ($\beta_{0}>0$), the integral of the Equation (11) can be calculated analytically by a change of variables for the integration. Consider a new variable $\varepsilon :=E_{x}\left(\tau \right)$, now, since the strain rate is constant, we have that $\varepsilon =E_{x}\left(\tau \right)=\beta_{0}\tau$, so the change of variables is given by:

$$E_{x}\left(\tau \right)=\varepsilon ,\;\tau =\varepsilon /\beta_{0},\;{\dot{E}}_{xx}d\tau =\beta_{0}d\tau =d\varepsilon$$

Introducing this change into the Equation (11), we obtain an explicit form for the integral which gives the viscoelastic contribution:

$$I_{k}=e^{-\frac{E_{x}}{\beta_{0}\tau_{k}}}\int_{0}^{E_{x}/\beta_{0}}e^{\frac{\varepsilon }{\beta_{0}\tau_{k}}}\left(1+2b_{0}\varepsilon^{2}\right)e^{b_{0}\varepsilon^{2}}\;d\varepsilon$$

By defining $\theta_{k}:=\beta_{0}\tau_{k}$, we can express it as:

$$I_{k}=E_{x}e^{b_{0}E_{x}^{2}}+\frac{e^{-\frac{E_{x}}{\theta_{k}}}-e^{b_{0}E_{x}^{2}}}{2\theta_{k}b_{0}}+\frac{e^{-\left(\frac{E_{x}}{\theta_{k}}+\frac{1}{4\theta_{k}^{2}b_{0}}\right)}}{4\theta_{k}^{2}b_{0}^{3/2}}F_{v}\left(E_{x},b_{0},\theta_{k}\right)$$

where the function $F_{v}\left(E_{x},b_{0},\theta_{k}\right)$ has been defined to abbreviate the notation and is given by:

$$F_{v}\left(E_{x},b_{0},\theta_{k}\right):=\sqrt{\pi }i\left[\mathrm{erf}\left(\frac{i}{2\theta_{k}b_{0}^{1/2}}\right)-\mathrm{erf}\left(+\frac{i}{2\theta_{k}b_{0}^{1/2}}+iE_{x}b_{0}^{1/2}\right)\right]$$

This function $F_{v}$ is a real function of real variable, despite the explicit appearance of $i=\sqrt{-1}$, since here, erf(·) represents the *error function*, which is given by:

$$\mathrm{erf}\left(x\right):=\frac{2}{\sqrt{\pi }}\int_{0}^{x}e^{-z^{2}}dz\;\Rightarrow \;i\cdot \mathrm{erf}\left(ix\right)=-\frac{2}{\sqrt{\pi }}\sum_{k=0}^{\infty }\frac{x^{2k+1}}{(2k+1)k!}\in R,\;\forall x\in R$$

The axial stress is given by:

(A1) $$S_{x}=b_{0}c\left[E_{x}e^{b_{0}E_{x}^{2}}+\sum_{k=1}^{N}g_{k}\left(E_{x}e^{b_{0}E_{x}^{2}}+\frac{e^{-\frac{E_{x}}{\theta_{k}}}-e^{b_{0}E_{x}^{2}}}{2\theta_{k}b_{0}}+\frac{e^{-\left(\frac{E_{x}}{\theta_{k}}+\frac{1}{4\theta_{k}^{2}b_{0}}\right)}}{4\theta_{k}^{2}b_{0}^{3/2}}F_{v}\left(E_{x},b_{0},\theta_{k}\right)\right)\right]$$

The effect of strain rate on the above equation was shown in Figure 7 from which it follows that for small strains, Equation (A1) produces only small changes; in particular, the stiffness or tangent Young’s modulus $dS_{x}/dE_{x}\to b_{0}c\left(1+g_{1}+dots+g_{N}\right)$ tends to the same value for $E_{x}\to 0$ regardless of the strain rate, as was shown in Figure 8, which was obtained by deriving Equation (A1).

*General case*. In the case of very high-speed testing, a conventional UTM will generally not be able to bring the specimen to the desired strain rate instantaneously but will require an acceleration time. Since constitutive parameter settings generally require knowledge of the analytical form, it is important to have the means to obtain such a formula or a reasonable approximation of it.

For this purpose, it is sufficient to note that since any continuous curve in sections can be approximated to any desired degree of accuracy by a polynomial, it is useful in viscoelastic calculations to evaluate integrals of the form:

(A2) $$\int_{0}^{t}e^{-\frac{t-\tau }{\tau_{0}}}P_{n}\left(\tau \right)\;d\tau$$

where $P_{n}\left(t\right)=\alpha_{n}t^{n}+\cdots +\alpha_{1}t+\alpha_{0}$ is a polynomial of degree *n*. The above integral can be evaluated using the formula:

$$\int_{0}^{t}e^{\frac{\tau }{\tau_{0}}}\tau^{m}\;d\tau =\tau_{0}e^{\frac{t}{\tau_{0}}}\left[t^{m}-mt^{m-1}\tau_{0}+\frac{m(m-1)}{2}t^{m-2}\tau_{0}^{2}-\cdots {\left(-1\right)}^{m}m!\tau_{0}^{m}\right]$$

where m∈N. The standard integral (Equation (A2)) can be expressed in the form:

∫0te−t−ττ0(αnτn+⋯+α0)dτ=∑s=0nαs∑r=0sr!sr(−1)rts−rτ0r+1+e−tτ0∑s=0nαs(−1)s+1s!τ0s+1

Using the confluent hypergeometric function ${}_{2}F_{0}\left(\left[a,b\right],\left[\;\right];x\right)$ [55,56], the first summand can be shortened:

$$\int_{0}^{t}e^{-\frac{t-\tau }{\tau_{0}}}\left(\alpha_{n}\tau^{n}+\cdots +\alpha_{0}\right)d\tau =\tau_{0}\sum_{s=0}^{n}\left[\alpha_{s}t^{s}\;{}_{2}F_{0}\left(\left[-s,1\right],\left[\;\right],\frac{t}{\tau_{0}}\right)\right]+e^{-\frac{t}{\tau_{0}}}\sum_{s=0}^{n}\alpha_{s}s!{\left(-\tau_{0}\right)}^{s+1}$$

The calculation for complex load curves can lead to long and cumbersome expressions, but the above formulas potentially allow a numerical approximation as accurate as desired for any load case.
